# Correlation of Fasting Blood Sugar and Glycated Hemoglobin (HbA1c) With Thiamine Levels in Diabetic Patients

**DOI:** 10.7759/cureus.46178

**Published:** 2023-09-29

**Authors:** Muhammad Ulusyar Khan, Muhammad Mubeen, Hira Khalid Chohan, Sidra Jawed, Aisha Jamal, Javeria Ahmed Qamar, Musarat Khalid Chohan, Ahsan Ali Siddiqui, Adnan Anwar, Atif A Hashmi

**Affiliations:** 1 Internal Medicine, Bolan University of Medical and Health Sciences, Quetta, PAK; 2 Internal Medicine, Dow University of Health Sciences, Karachi, PAK; 3 General Surgery, Dow University of Health Sciences, Karachi, PAK; 4 Internal Medicine, Jinnah Medical and Dental College, Karachi, PAK; 5 Internal Medicine, Army Medical College, Rawalpindi, PAK; 6 Anaesthesiology, Jinnah Sindh Medical University, Karachi, PAK; 7 Family Medicine, Tumair General Hospital, Riyadh, SAU; 8 Physiology, Hamdard College of Medicine and Dentistry, Karachi, PAK; 9 Internal Medicine, Essa General Hospital, Karachi, PAK; 10 Pathology, Liaquat National Hospital and Medical College, Karachi, PAK

**Keywords:** hba1c, glycated hemoglobin, diabetes mellitus type 1, diabetes mellitus type 2, thiamine level

## Abstract

Introduction

It has been discovered that low levels of thiamine reserves in the body are related to diabetes mellitus (DM) because thiamine directly influences carbohydrate metabolism. Therefore, the purpose of this study was to assess several metabolic variables and blood thiamine levels in patients with type 1 and type 2 DM and compare them with those in a control group of healthy individuals.

Methods

This case-control study was conducted at multiple diabetic outpatient centers in Karachi. A total of 90 participants, who were divided into three groups, each containing 30 individuals, were chosen using a convenient non-probability sampling technique. Group A served as the control group and consisted of healthy, non-diabetic individuals. Groups B and C contained subjects with type 1 and type 2 DM, respectively. Descriptive analysis was reported as mean standard deviation, whereas gender and comorbidities were expressed as frequencies and percentages. The chi-square test and Pearson’s correlation coefficient were used to determine the associations of the variables with type 1 DM, type 2 DM, and controls.

Results

The study results revealed statistically significant differences between controls, type 1 and type 2 DM, in the means of blood glucose levels and all lipid profiles, such as glycated hemoglobin (HbA1c), fasting blood sugar (FBS), random blood sugar (RBS), serum thiamine, triglycerides (p < 0.001), high-density lipoprotein (HDL) (p = 0.014), and total cholesterol (p = 0.013). Furthermore, it was shown that among the control group, type 1 and type 2 DM, HbA1c, and FBS were insignificantly correlated with thiamine levels, whereas the HbA1c and FBS of the combined diabetic groups were significantly correlated with the thiamine level (r = 0.465, p < 0.001) and (r = 0.360, p = 0.005), respectively, where 'r' is the Pearson correlation coefficient. Additionally, HbA1c and FBS in the combined three groups were significantly correlated with the thiamine level (r = −0.626, p < 0.001) and (r = −0.561, p < 0.001), respectively.

Conclusion

This study concluded that patients with type 1 and type 2 DM had significantly higher levels of FBS, RBS, HbA1c, triglycerides, and total cholesterol than controls. Furthermore, both type 1 and type 2 DM patients’ serum thiamine and HDL levels were observed to be considerably lower than those of controls. Additionally, among both types of DM and controls, there was a strong correlation between FBS and HbA1c. Therefore, we recommend that serum thiamine levels be routinely monitored in diabetic patients, and thiamine supplementation should be considered to avoid complications, especially vascular complications of DM.

## Introduction

Thiamine, generally known as vitamin B1, is a water-soluble vitamin that plays an important role in the metabolism of both vital amino acids and carbohydrates [1]. Thiamine has anti-inflammatory and antioxidant characteristics that affect endothelial function. In addition to free thiamine, it can also be found as thiamine diphosphate (TDP), thiamine monophosphate (TMP), thiamine triphosphate (TTP), and adenosine thiamine triphosphate (ATT). Thiamine is thought to be crucial for child development and plays an essential role in lipid metabolism [2]. For adult men and women, the recommended daily amounts of thiamine are 1.0-1.4 mg and 0.8-1.1 mg, respectively [3]. The thiamine transport protein system is primarily responsible for thiamine absorption in the duodenum. Thiamine is transported and delivered into tissues by two transporters, thiamine transporter 1 (THTR1) and thiamine transporter 2 (THTR2) [4]. The placenta, liver, and kidney are among the tissues that contain high levels of THTR1 and THTR2, but THTR1 is also present in skeletal tissues and cardiac muscles [5].

The level of thiamine in the body can be assessed using two methods: assessing the erythrocyte transketolase (ETK) assay's level of TDP capability and counting the amount of thiamine metabolites present in surrounding tissues. Because of its clear demonstration of vitamin functioning, the ETK assay is thought to be more beneficial [6,7]. Consequently, serum and urine thiamin levels are evaluated by blood testing and the measurement of the presence of thiamine metabolites in the urine, respectively [8].

One of the most serious medical concerns is diabetes mellitus (DM), whose prevalence is rising rapidly across all age categories. Genetic, external, and host variables are associated with this disease. DM can develop through two fundamental processes: autoimmune and metabolic mechanisms. Malabsorption, overweight, inactivity, and hormone imbalances are the main risk factors for this condition. DM is linked to insulin shock, renal failure, atherosclerosis, and stroke. The best approach to avoiding DM, especially type 2, is through consistent physical activity and a healthy diet [9,10]. The two main classifications of DM are type 1 (insulin-dependent DM) and type 2 (insulin-independent DM). Type 1 DM is caused by the inability of pancreatic beta cells to make insulin and is more frequent than type 2 DM, which is caused by insulin resistance and reduced insulin receptor sensitivity [9]. In underdeveloped nations such as Pakistan, type 2 DM is becoming more widespread [10]. Because thiamine directly influences the metabolism of carbohydrates, DM is associated with low levels of thiamine reserves in the body [11].

Both types 1 and 2 DM have been linked to decreased thiamine levels and increased renal clearance [12]. A cross-sectional comparison investigation clearly demonstrated this with healthy controls and DM patients with microalbuminuria and macroalbuminuria. DM has been linked to low levels of thiamine as well as a gradual decline in albumin levels in the urine, particularly in cases of macroalbuminuria. Microalbuminuria also showed a negative correlation between thiamin level and lipid profile [13].

However, there is still uncertainty regarding the relationship between thiamine intake and conditions such as DM, cerebrovascular diseases (CVDs), aberrant cholesterol levels, and behavioral wellness. In addition, thiamine supplements have been suggested to be helpful in patients with type 2 DM to avoid renal and cardiovascular diseases, improve quality of life, and reduce consequences [14]. Therefore, the aim of this study was to compare patients with type 1 and type 2 DM with healthy controls to examine numerous demographic characteristics and variations in serum thiamine levels.

## Materials and methods

This was a case-control study conducted at multiple diabetic outpatient centers in Karachi. The ethical approval of the study was obtained from Essa General Hospital (Essa/16/2022). The study lasted for six months, from February 2022 to July 2022. A total of 90 participants, which were divided into three groups, each containing 30 individuals, were chosen using a convenient non-probability sampling method. Group A served as the control group and consisted of medically fit individuals. Groups B and C contained individuals with type 1 and type 2 DM, respectively. The study included all individuals with type 1 and type 2 DM of both genders between the ages of 25 and 46. However, individuals on diuretics, those with serious concurrent medical conditions such as gastrointestinal illness, end-stage renal disease, coronary artery disease, and chronic liver disease, or those who had undergone transplant surgical procedures, were excluded from the study.

Demographic information, including gender, age, and concurrent illnesses, was recorded after the participants provided informed consent. Blood samples were obtained in heparinized tubes from multiple diabetic clinics. Blood samples collected in a non-heparinized tube were promptly centrifuged for 20 minutes at 2000 rpm. The evaluation of numerous biochemical diagnostic factors, including creatinine, urea, random blood sugar (RBS), fasting lipid profile, thiamine levels in blood and urine, and microalbuminuria, was performed using the clear serum supernatant. In both sitting and standing positions, the blood pressure of the right arm was checked twice. Averaging two readings from measurements conducted five minutes apart was recorded for all individuals.

Data analysis was performed using the Statistical Package for Social Science (SPSS, Version 26.0; IBM, Inc., Armonk, NY, USA). Descriptive statistics are reported as means and standard deviations, whereas gender and comorbidities are expressed as frequencies and percentages. The one-way analysis of variance (ANOVA) was applied to associate the means among the three study groups. The chi-square test and Pearson’s correlation coefficient were used to determine the associations of variables with type 1 and type 2 DM and controls. A p-value < 0.05 was considered statistically significant.

## Results

A total of 90 participants, with 30 participants each in the control, type 1 DM, and type 2 DM groups, were included in the study. In the control group, half of the participants -15 (50.0%)- were males and 15 (50.0%) were females. In type 2 DM, 22 (73.3%) participants were males and 8 (26.7%) were females. In contrast, 20 (66.7%) males and 10 (33.3%) females had type 2 DM, with a significant difference among them (p = 0.155). Comorbidities revealed that hypertension, neuropathy, and coronary artery disease were significantly associated among controls and type 1 and 2 DM participants (p < 0.05), as presented in Table 1.

**Table 1:** Demographic details of type 1 and type 2 diabetes mellitus patients and healthy controls (n=90) DM: diabetes mellitus. *p-value significant as <0.05.

Variables			n	%	p-value
Gender	Male	Control	15	50.0	0.155
		Type 1 DM	22	73.3	
		Type 2 DM	20	66.7	
	Female	Control	15	50.0	
		Type 1 DM	8	26.7	
		Type 2 DM	10	33.3	
Hypertension	Yes	Control	2	6.7	<0.001*
		Type 1 DM	11	36.7	
		Type 2 DM	19	63.3	
	No	Control	28	93.3	
		Type 1 DM	19	63.3	
		Type 2 DM	11	36.7	
Neuropathy	Yes	Control	0	0	0.003*
		Type 1 DM	2	6.7	
		Type 2 DM	8	26.7	
	No	Control	30	100.0	
		Type 1 DM	28	93.3	
		Type 2 DM	22	73.3	
Coronary artery disease	Yes	Control	0	0	<0.001*
		Type 1 DM	2	6.7	
		Type 2 DM	10	33.3	
	No	Control	30	100.0	
		Type 1 DM	28	93.3	
		Type 2 DM	20	66.7	

The association among controls, type 1 and type 2 DM, revealed statistically significant differences in all basic demographics, including age (p < 0.001), body mass index (BMI) (p < 0.001), diabetes duration (p<0.001), systolic blood pressure (p = 0.001), diastolic blood pressure (p = 0.002), and heart rate (p < 0.001), as presented in Table 2.

**Table 2:** The association of demographics variables and vitals among controls and type 1 and 2 diabetes mellitus patients DM: diabetes mellitus. *p-value significant as <0.05.

Variables		Mean	Standard deviation	p-value
Age (years)	Control	41.73	6.88	<0.001*
	Type 1 DM	24.20	6.397	
	Type 2 DM	42.73	10.51	
Body mass index (kg/m^2^)	Control	28.55	7.67	<0.001*
	Type 1 DM	15.63	2.87	
	Type 2 DM	31.85	5.63	
Duration of DM (years)	Control	0	0	<0.001**
	Type 1 DM	4.07	1.94	
	Type 2 DM	8.93	2.80	
Blood pressure systolic (mm Hg)	Control	122.0	10.30	0.001*
	Type 1 DM	129.67	12.99	
	Type 2 DM	133.67	12.72	
Blood pressure diastolic (mm Hg)	Control	81.0	8.847	0.002*
	Type 1 DM	89.67	13.51	
	Type 2 DM	91.0	10.93	
Heart rate (beats per minute)	Control	71.63	4.97	<0.001*
	Type 1 DM	77.30	8.65	
	Type 2 DM	78.73	5.57	

Furthermore, the association among controls, type 1 and type 2 DM, revealed statistically significant differences in the means of blood glucose levels and all lipid profiles such as glycated hemoglobin (HbA1c), fasting blood sugar (FBS), RBS, serum thiamine, triglycerides (p < 0.001), high-density lipoprotein (HDL) (p = 0.014), and total cholesterol (p = 0.013), except low-density lipoprotein (LDL) (p = 0.237), as presented in Table 3.

**Table 3:** The association of blood glucose level and lipid profile among controls and type 1 and type 2 diabetes mellitus patients DM: diabetes mellitus; HbA1c: glycated hemoglobin. *p-value significant as <0.05.

Variables		Mean	Standard deviation	p-value
HbA1c (%)	Control	5.20	0.29	<0.001*
	Type 1 DM	7.49	0.62	
	Type 2 DM	9.38	1.97	
Fasting blood sugar (mg/dl)	Control	87.10	11.78	<0.001*
	Type 1 DM	151.30	46.03	
	Type 2 DM	211.77	72.13	
Random blood sugar (mg/dl)	Control	146.50	28.96	<0.001*
	Type 1 DM	268.33	36.54	
	Type 2 DM	282.50	45.55	
Serum thiamine	Control	69.55	12.75	<0.001*
	Type 1 DM	7.34	1.90	
	Type 2 DM	14.89	4.82	
Triglycerides (mg/dl)	Control	117.43	18.76	<0.001*
	Type 1 DM	169.43	57.25	
	Type 2 DM	152.17	56.96	
Low-density lipoprotein (mg/dl)	Control	110.05	22.13	0.237
	Type 1 DM	118.13	16.11	
	Type 2 DM	113.63	16.08	
High-density lipoprotein (mg/dl)	Control	45.63	7.37	0.014*
	Type 1 DM	40.40	6.70	
	Type 2 DM	41.87	6.85	
Total cholesterol (mg/dl)	Control	177.83	16.14	0.013*
	Type 1 DM	202.20	34.19	
	Type 2 DM	189.27	38.60	

The study results showed that among the control group, type 1 and type 2 DM, HbA1c, and FBS were insignificantly correlated with thiamine levels, whereas the HbA1c and FBS of the combined diabetic groups were significantly correlated with the thiamine level (r = 0.465, p < 0.001) and (r = 0.360, p = 0.005), respectively, where 'r' is the Pearson correlation coefficient. Additionally, the HbA1c and FBS of the combined three groups were significantly correlated with the thiamine level (r = −0.626, p < 0.001) and (r = −0.561, p < 0.001), respectively, as presented in Table 4.

**Table 4:** The correlation of fasting blood sugar and glycated hemoglobin with thiamine levels in controls and diabetic patients r: Pearson correlation coefficient; DM: diabetes mellitus; HbA1c: glycated hemoglobin; FBS: fasting blood sugar. *p-value significant as <0.05.

Groups	Variables	Serum thiamine level	
		r	p-value
Group A (control) (n=30)	HbA1c	0.1	0.598
	FBS	−0.12	0.527
Group B (type 1 DM) (n=30)	HbA1c	0.072	0.706
	FBS	0.074	0.697
Group C (type 2 DM) (n=30)	HbA1c	0.117	0.538
	FBS	0.05	0.794
Combined diabetic groups (n=60)	HbA1c	0.465	<0.001*
	FBS	0.360	0.005*
Combined three groups (n=90)	HbA1c	−0.626	<0.001*
	FBS	−0.561	<0.001*

## Discussion

Increased renal thiamine clearance and moderate thiamine deficiency are frequently observed in diabetic individuals [15]. Therefore, this study demonstrated the relationship between diabetics and healthy controls with variations in thymine levels.

One study revealed that people with type 1 DM had considerably lower serum thiamine levels than those with type 2 DM and healthy controls [16]. Thiamine levels in patients with type 1 DM were significantly lower than those in controls in a previous study [17]. These findings were corroborated with the results of our study and revealed that blood thiamine levels were substantially lower in type 1 (7.34 ± 1.90) and type 2 (14.89 ± 4.82) DM patients than in controls (69.55 ± 12.75) (p < 0.001).

Surprisingly, another study showed that patients with type 1 DM had blood thiamine levels that were much lower than those of healthy controls and that there was an inverse link between thiamine and blood glucose levels [18]. Another study revealed that 18 (60%) type 1 DM patients had blood thiamine levels that were considerably lower than those of healthy controls [19]. This leads to the conclusion that thiamine eventually contributes to diabetic endothelium vascular disorders (micro and macroangiopathy), lipid disorders, kidney failure, retinal degeneration, cardiopathy, and neuropathic illnesses. Our study was inconsistent with the above-reported research and revealed that the FBS level and HbA1c in type 1 diabetes had an insignificant association and correlation with the thiamine levels (r = 0.072, p = 0.706 and r = 0.074, p = 0.697, respectively).

One of the investigations conducted at a rural medical college was to determine the pattern of dyslipidemia in individuals with type 2 DM. Increased triglycerides in the blood were observed in 56% of patients, and low HDL, which was discovered in 52.9% of cases, was the most often detected dyslipidemia [20]. In a comparable manner, a different study revealed that low HDL levels were the dyslipidemia most frequently seen in patients with type 2 DM (r = 0.454, p = 0.012), even though triglyceride levels were also elevated but not to a clinically significant degree [21]. These findings were consistent with those of the present study and showed that triglyceride levels were substantially higher in patients with type 1 and type 2 DM than in controls (p < 0.001). Likewise, lower HDL levels were significantly reported in type 1 and type 2 DM patients than in the controls (p = 0.014).

Another cross-sectional study discovered an association between blood glucose levels and serum lipid parameters, showing that early detection of lipid irregularities can reduce the probabilities of cardiovascular illness and cerebrovascular devastation in type 2 DM participants. In addition, it was noted that there was an insignificant link between HbA1c and LDL in patients with type 2 DM, but there was a significant correlation between HbA1c and blood levels of triglycerides, total cholesterol, and HDL (p < 0.05) [22]. These results were in line with another study's findings, which showed a significant correlation between HDL and systolic blood pressure in patients with type 2 DM (r = 0.454, p = 0.012), but an insignificant correlation between total cholesterol, triglyceride, and LDL levels [21]. Our findings were not in accordance with the abovementioned studies, which indicated that FBS, RBS, and HbA1c levels were significantly associated with type 1 and type 2 DM (p < 0.001).

Another comparative study assessed thiamine levels in patients with type 1 and type 2 DM. They discovered that these patients had considerably higher RBS, FBS, and HbA1c levels than those in the control group. In addition, patients with type 1 and type 2 DM had higher triglyceride and total cholesterol levels than those in the control groups. The study findings also showed that individuals with type 1 and type 2 DM had considerably reduced mean HDL levels compared with those in the control group. One interesting conclusion of their research was that patients with type 1 and type 2 DM had significantly lower mean serum thiamine levels than those without DM [23], a conclusion that is consistent with another study. According to a previous study conducted in 2015, individuals with type 1 DM had considerably higher glucose levels than controls (p = 0.001) [19]. Likewise, according to research, the HbA1c levels of patients with type 1 and type 2 DM were considerably higher than those of controls [23]. An earlier investigation from 2003 found that people with DM had higher HbA1c levels than healthy non-diabetic participants (p = 0.002). Additionally, HbA1c has been proposed as a very accurate and practical screening and diagnostic method for DM [24]. In addition, a study indicated that triglyceride and total cholesterol levels were significantly higher in patients with type 1 and type 2 DM than in healthy participants. Moreover, triglyceride and cholesterol levels have been previously reported to be significantly greater in patients with type I DM than in healthy participants (p = 0.008) [17]. These findings were consistent with those of the present study and revealed that RBS, FBS, and HbA1c levels were significantly higher in patients with type 1 and type 2 DM than in healthy participants (p < 0.001). In addition, lipid profiles, such as triglycerides and total cholesterol, were significantly higher in type 1 and type 2 DM than in healthy participants (p < 0.05), whereas HDL was significantly reduced in type 1 and type 2 DM (p = 0.014). LDL levels were insignificantly increased in patients with type 1 and type 2 DM and healthy participants (p = 0.237).

The results from another study also showed that type 1 and type 2 DM patients had considerably lower serum thiamine levels than non-diabetic participants [23]. It was also reported in a study conducted in 2016 that thiamine levels were considerably lower in patients with type 1 DM than in controls (p = 0.002) [17]. Another study discovered that patients with type 1 and type 2 DM had substantially lower plasma thiamine concentrations than healthy controls (p < 0.001 for both) [15]. Similar findings were found in a 2012 study that found that individuals with DM types 1 and 2 had substantially lower blood thiamine concentrations than controls (p < 0.001) [11]. Our study showed similarity to the abovementioned research and indicated that thiamine levels were significantly lower in patients with type 1 and type 2 DM than in controls (p < 0.001).

This study had a few limitations. Owing to observer bias and a non-probability sampling method, this study may contain selection bias. Therefore, future studies using the probability sampling method are advised to explore this relationship with greater numbers of samples to obtain more accurate results. Furthermore, although it was a multicenter study, the sample size was limited. Moreover, long-term follow-up was not performed in our study to correlate diabetic complications with serum thiamine levels.

## Conclusions

This study concluded that patients with type 1 and type 2 DM had significantly higher levels of FBS, RBS, HbA1c, triglycerides, and total cholesterol than controls. Furthermore, the serum thiamin and HDL levels of patients with type 1 and type 2 DM were observed to be considerably lower than those of controls. Additionally, among both types of DM and controls, there was a strong correlation between FBS and HbA1c levels. High blood sugar levels in DM may lead to increased thiamine excretion, resulting in thiamine deficiency, which may augment the diabetic complications, especially the vascular complications of DM. Therefore, we recommend that thiamine levels be routinely monitored in diabetic patients, and early thiamine supplementation should be considered, especially if there are signs of vascular complications on clinical examination.
